# From Emissions to Assets: Sustainable Technologies for CO_2_ Capture, Conversion, and Integrated Strategies

**DOI:** 10.3390/ijms27020847

**Published:** 2026-01-14

**Authors:** Shokouh Masoumilari, Zohreh Masoumi, Alireza Mahvelati Shamsabadi, Daeseung Kyung, Meysam Tayebi

**Affiliations:** Department of Civil and Environment Engineering, University of Ulsan, Daehakro 93, Namgu, Ulsan 44610, Republic of Korea; shokoohmasoumi2@gmail.com (S.M.); zohrehmasoumi17@gmail.com (Z.M.); a.mahvelati@hotmail.com (A.M.S.)

**Keywords:** carbon capture and utilization, CO_2_ conversion, environmental impacts, formic acid, methanol

## Abstract

Addressing the growing threat of climate change requires urgent and sustainable solutions for managing carbon dioxide (CO_2_) emissions. This review investigates the latest advancements in technologies for capturing and converting CO_2_, with a focus on approaches that prioritize energy efficiency, environmental compatibility, and economic viability. Emerging strategies in CO_2_ capture are discussed, with attention to low-carbon-intensity materials and scalable designs. In parallel, innovative CO_2_ conversion pathways, such as thermocatalytic, electrocatalytic, and photochemical processes, are evaluated for their potential to transform CO_2_ into valuable chemicals and fuels. A growing body of research now focuses on integrating capture and conversion into unified systems, eliminating energy-intensive intermediate steps like compression and transportation. These integrated carbon capture and conversion/utilization (ICCC/ICCU) technologies have gained significant attention as promising strategies for sustainable carbon management. By bridging the gap between CO_2_ separation and reuse, these sustainable technologies are poised to play a transformative role in the transition to a low-carbon future.

## 1. Introduction

The atmospheric CO_2_ concentration has increased by over 50% since the Industrial Revolution, surpassing 420 ppm and threatening climate stability. Projections estimate CO_2_ levels could reach 570 ppm by 2100, potentially causing a 3.2 °C global temperature rise [1,2]. Most emissions stem from point sources like power plants, making effective carbon management strategies crucial [3,4].

Carbon capture, utilization, and storage (CCUS) can help address climate issues by capturing CO_2_ from specific sources or directly from the atmosphere [5]. Carbon capture, utilization, and storage (CCUS) methods, including chemical looping and catalytic conversion, are essential to reduce emissions [6,7].

The global CCUS goal is to achieve a carbon-negative future society by reducing CO_2_ emissions and producing sustainable fuels and chemicals through capturing CO_2_ and converting to high value-added compounds [8,9]. Converting CO_2_ through photochemical and electrochemical reduction reactions (CO_2_RR) shows potential in creating valuable products, leveraging sustainable as well as renewable energy sources [10,11]. Both technologies have shown the capability and potential for generating the fuels which are considered as being both clean and emission-free, and they present significant benefits and power in addressing global warming in addition to reducing anthropogenic CO_2_ emissions [12,13,14].

Researchers have developed various methods to study and evaluate the catalytic reduction of CO_2_ toward its industrial application [15]. System modeling plays a vital role in understanding and optimizing CO_2_ capture and conversion processes [16]. These models help researchers and engineers design and develop efficient systems for photo/catalytic CO_2_ capture and conversion [17]. Additionally, life cycle assessment (LCA) is an environmental assessment technique that evaluates the ecological effects of a process or product from the time raw materials are first extracted until the end of its useful life, when it is disposed of [18,19]. Catalytic CO_2_ reduction methods are under study for industrial applications, supported by system modeling and life cycle assessment (LCA) to evaluate sustainability and ecological impacts, aiding in developing efficient CO_2_ capture and conversion technologies [20,21,22,23,24].

This review addresses this evolving landscape by providing a unified, system-level analysis of sustainable CO_2_ management. Unlike prior reviews that focus on isolated components (capture, catalysis, or conversion), this work integrates these domains to critically assess the transition from standalone processes to integrated systems. We adopt a cross-cutting life-cycle and techno-economic framework to compare the scalability, cost, and holistic sustainability of competing pathways, with a particular focus on the production of formic acid and methanol as key value-added products. By examining advances in multifunctional materials, reactor design, and renewable energy coupling, this review aims to provide a holistic roadmap for the development of scalable, commercially viable ICCU technologies capable of supporting a circular carbon economy.

## 2. Sustainable CO_2_ Capture and Conversion

The high levels of CO_2_ emissions lead to environmental issues which means applying sustainable management strategies is crucial for carbon capture, utilization, and storage (CCUS), so that we can handle the challenges associated with emission control [25,26,27]. In approaches to CO_2_ capture and conversion processes, there exist potential benefits for saving energy by combining them effectively to skip the need for energy-intensive steps including regeneration and transportation involved in the traditional sequential pathway [28,29,30]. The integrated capturing and converting carbon (ICCC/ICCU) technology is considered efficient and sustainable for capturing and utilizing CO_2_. It shows efficiency in converting CO_2_ and helps reduce costs by eliminating the need for compressing and transporting CO_2_.

**Figure 1:**
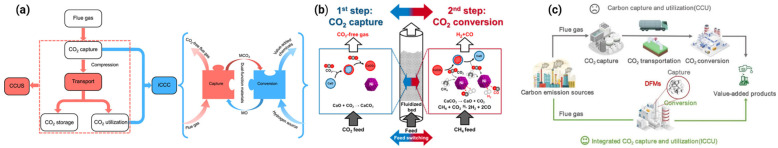
(**a**) Diagrammatic representations that contrast the development of (ICCC/ICCU) technologies with that of conventional CCUS technologies [31]. (**b**) A schematic illustration of the proposed method that incorporates dry reforming of methane (DRM) directly into the CO_2_ collection process, resulting in synthesis gas [32]. (**c**) Schematic diagrams of CCU and ICCU processes [33].

Traditional carbon capture and utilization (CCU) technologies, as shown in Figure 1a (left), involve multiple sequential steps, including CO_2_ capture, adsorbent regeneration, compression, transportation, and finally, conversion or storage. These multistep processes are not only energy-intensive but also cause significant material degradation, reducing overall process efficiency. To overcome these limitations, an integrated CO_2_ capture and utilization (ICCU) approach has been proposed, as depicted in Figure 1a (right) and further detailed in Figure 1c. In ICCU, CO_2_ is captured and directly converted into valuable products within a single reactor using dual-function materials (DFMs), eliminating the need for intermediate steps like compression and transport. A specific example of this is the calcium looping-based ICCU process shown in Figure 1b, where CaO captures CO_2_ to form CaCO_3_ in the first step. In the second step, under a methane (CH_4_) atmosphere, the CaCO_3_ is regenerated back to CaO while the released CO_2_ simultaneously undergoes dry reforming of methane (DRM) to produce synthesis gas (syngas: CO + H_2_). This integrated method greatly improves energy efficiency and system simplicity, offering a promising route for sustainable CO_2_ management.

Carbon-containing compounds like methane (CH_4_) methanol (CH_3_OH) and formic acid (HCOOH) show potential for CCU. This new method enables the storage of energy while also capturing CO_2_ at the same time, a dual benefit making it different from traditional approaches. A key advantage of this technology is its ability to produce CH_4_ from gas by using a nickel-based catalyst known as the Sabatier reaction, in the process of CO (or CO_2_) hydrogenation [34].

Furthermore, the evaluation of CO_2_ value chains over their life cycle has traditionally relied on ad hoc criteria, such as the quantity of CO_2_ utilized and captured, and simplified CO_2_ balances. A comprehensive evaluation of the life cycle can serve as a basis for assessing the sustainability of CO_2_ value chains, considering environmental and economic factors. Utilizing optimization methods can be beneficial for evaluating the sustainability of CO_2_ value chains [31].

### Sustainability Criteria and Strategic Role of Integrated CO_2_ Capture–Conversion Technologies

Sustainability in CO_2_ capture, conversion, and utilization requires a holistic framework that considers energy efficiency, material sustainability, resource intensity, environmental safety, and integration. Although renewable pathways may have higher current energy demands, they offer long-term sustainability through compatibility with low-carbon energy. Integrated capture and conversion strategies enhance sustainability by minimizing energy penalties and enabling decentralized processes. High-TRL systems are suited for centralized sectors, while emerging electrochemical pathways are better for flexible applications. Sustainable CO_2_ utilization focuses on resilience, scalability, and environmental compatibility rather than just immediate efficiency.

## 3. CO_2_ Capture

Carbon capture is a process involving the capture of CO_2_ emissions from sources like power plants and industrial sites, or even directly from the air itself, utilizing specialized technology-induced methods such as pre-combustion capture (capturing CO_2_ before fuel burning) post-combustion capture (capturing CO_2_ after fuel burning), oxy-fuel combustion (burning fuel, in pure oxygen rather than air), and direct air capture (extracting CO_2_ directly from the atmosphere) [35,36,37].

Different technologies are used for capturing CO_2_, such as direct air capture (DAC), absorption (CO_2_ is absorbed by a solvent and then released for storage), adsorption (CO_2_ is captured by solid materials), and membrane separation (CO_2_ is separated from other gases using a semi-permeable membrane) [38,39]. Once captured, CO_2_ needs to be transported to storage sites, usually through pipelines or, in some cases, by truck or ship [40].

While conventional CO_2_ capture technologies such as amine scrubbing, solid sorbents, and membrane separation remain the foundation for industrial-scale carbon management, the integration of capture and conversion (ICCC/ICCU) represents the most innovative and rapidly developing direction. Integrated systems avoid the energetic penalties associated with CO_2_ release, purification, and compression, enabling direct utilization of captured CO_2_ under mild conditions. This approach provides significant advantages in energy efficiency, system compactness, and overall process economics. Therefore, the following section expands on ICCC/ICCU concepts in greater detail, reflecting their growing importance and disruptive potential in next-generation carbon utilization technologies.

### Molecular Design and Structure–Property Relationships in CO_2_ Capture Materials

The molecular and structural characteristics of CO_2_ capture materials directly dictate their performance and stability. In amine-based solvents, the specific chemistry between the amine group and CO_2_, influenced by factors like steric hindrance (e.g., in AMP), determines whether carbamate or bicarbonate forms, which in turn governs the energy-intensive regeneration step. For solid sorbents like Metal–Organic Frameworks (MOFs), their exceptional surface area and tunable pores are ideal for physisorption [41], but their practical application is often limited by poor hydrolytic stability [42], a challenge that can be addressed through molecular design such as incorporating hydrophobic linkers. Conversely, high-temperature chemisorbents like CaO suffer from sintering—the loss of surface area and pore structure due to particle agglomeration. This macroscopic failure is mitigated at the atomic scale by doping with inert materials like Al_2_O_3_ or MgO, which create a thermally stable nanostructure to preserve the sorbent’s morphology and capacity over repeated capture–regeneration cycles [33]. Thus, optimizing CO_2_ capture relies on understanding and engineering these fundamental structure–property relationships at the molecular level.

**Figure 2:**
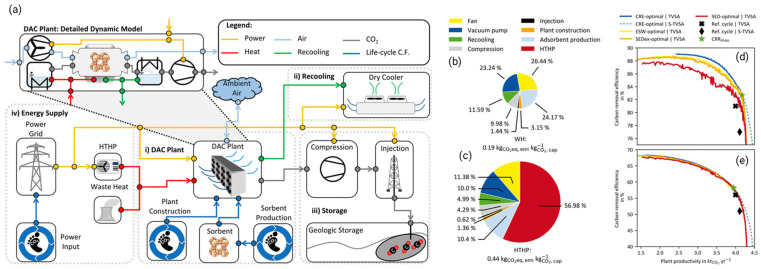
(**a**) The model scheme of the DACCS system is shown, with power flows marked in yellow, heat flows in red, and waste heat flows in green. The life cycle GHG emissions are shown with blue lines. (**b**) WH case and (**c**) HTHP case. Trade-offs between plant productivity and carbon removal efficiency for different KPI-optimal processes: (**d**) WH case and (**e**) HTHP case [43].

As shown in schematic at Figure 2a, Postweiler et al. [43] explored the optimization of adsorption-based Direct Air Carbon Capture and Storage (DACCS) systems, emphasizing the need for systemic climate-benefit metrics like Carbon Removal Efficiency (CRE) and Carbon Removal Rate (CRR) over traditional energy-related Key Performance Indicators (KPIs) such as specific energy demand (SED) and equivalent shaft work (ESW). Figures illustrate that using CRE as a KPI can enhance both carbon removal efficiency and plant productivity, with notable shifts in Pareto frontiers toward higher values (Figure 2d,e). The study employs a dynamic DACCS model that incorporates life-cycle greenhouse gas emissions, enabling comprehensive process analysis (Figure 2b,c). It highlights the importance of cleaner energy sources, showing that lower GHG emissions from electricity significantly improve DACCS performance. The findings advocate flexible DACCS operations in response to varying electricity GHG emissions and recommend integrating CRE with economic assessments for broader applicability in negative emission technologies.

This critical assessment underscores that while Direct Air Capture (DAC) shows promise, its path to climate-relevant scale is constrained by profound systemic challenges. Its immense energy demand would compete for scarce renewable electricity, and its net benefit depends on a fully decarbonized grid—creating a circular dependency. Coupled with massive material and infrastructure requirements, these hurdles position DAC not as a replacement for aggressive source emission reductions, but as a necessary, high-cost complement for addressing distributed and legacy emissions.

**Figure 3:**
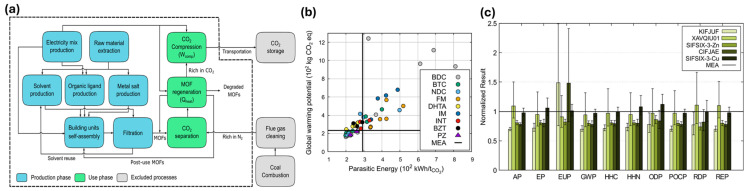
(**a**) The system boundary was chosen for the MOF-based CCS process’s LCA. Every block represents a distinct unit process. (**b**) The connection between parasitic energy and GWP. The horizontal and vertical lines, respectively, indicate the MEA-based CCS process’s GWP and parasitic energy. (**c**) The five best-performing MOFs were ranked by parasitic energy (normalized to MEA), with KIFJUF showing the lowest and SIFSIZ-3-Cu the highest, as illustrated with error bars indicating variability [44].

Hu et al. [44] evaluated the environmental impacts of Metal–Organic Frameworks (MOFs) for carbon capture after combustion as opposed to monoethanolamine (MEA) scrubbing technology using LCA and molecular simulations (Figure 3a). It highlights that solvent type and recycling rates are critical in determining the overall environmental impact, particularly affecting eutrophication potential (EUP). Figure 3b illustrates the relationship between parasitic energy, greenhouse gas emissions, and the performance of various MOFs, showing that some can achieve up to 33% lower energy consumption than MEA. As shown in Figure 3c, the study identifies five top-performing MOFs, including SIFSIX-3-Zn, which have lower environmental impacts than MEA, emphasizing the importance of optimizing synthesis conditions and using greener solvents.

Table 1 provides an overview of the main technologies for CO_2_ capture, highlighting their relative maturity, efficiency, and scalability. Pre-combustion capture is generally recognized for its efficiency, while post-combustion capture allows for retrofitting and modifications to existing systems. Oxy-fuel combustion enables the production of CO_2_-rich flue gas, facilitating easier separation, while direct air capture (DAC) is particularly notable for its potential to deliver negative emissions, despite its very high energy and cost requirements. Across all of these approaches, the major challenges remain the high initial capital costs, significant energy demand, and in some cases the costly production of oxygen, all of which may limit their broad deployment.

Amine-based absorption is currently the most mature and widely deployed option, with capture efficiencies of 85–95% and proven commercial readiness. However, its widespread use is hindered by the substantial energy penalty associated with solvent regeneration as well as issues such as solvent degradation and corrosion. Calcium looping represents another near-commercial pathway, offering comparable efficiency with relatively inexpensive sorbents, and is particularly well suited to high-temperature industries such as power generation and cement production. Emerging technologies, including solid sorbents and membranes, are attractive due to their lower energy requirements, modularity, and compact design, but remain challenged by moisture sensitivity, selectivity limitations, and questions of scalability. Cryogenic separation, while capable of producing extremely pure CO_2_, is energy-intensive and therefore restricted to niche applications where concentrated CO_2_ streams are already available. DAC stands out for its ability to capture CO_2_ from diffuse sources, enabling long-term climate strategies that rely on negative emissions. However, its future deployment depends heavily on breakthroughs in process efficiency and the integration of renewable energy to reduce costs. Biological pathways, such as algae cultivation and biochar formation, offer co-benefits including biomass production and soil enrichment, while requiring comparatively less energy. Nevertheless, their scalability is constrained by land and water availability as well as generally low CO_2_ uptake rates.

Despite high energy costs, Table 1 shows that mature CO_2_ capture technologies, such as calcium looping and amine absorption, are expected to lead to large-scale adoption because of their high readiness and efficiency. Solid sorbents, membranes, and direct air capture (DAC) are examples of emerging technologies that enable modular designs but have issues with energy cost and demand, suggesting a trade-off between their maturity and flexibility. There is no single technology that works best for everyone; instead, complementarity is what makes them useful. Amine absorption and calcium looping will predominate in the near future, but if costs come down in the medium to long term, additional technologies could become more popular. Furthermore, biological capture can offer synergies in larger sustainable systems despite its restricted use.

A critical synthesis of the CO_2_ capture and formic acid conversion literature reveals significant trade-offs in efficiency, scalability, and readiness. In capture, amine scrubbing remains the most mature (TRL 8–9) with high efficiency (85–95%) but suffers from high energy penalties and solvent degradation, whereas emerging solid sorbents and membranes offer modularity and lower energy use but face stability and selectivity challenges under realistic conditions [45]. Direct air capture enables negative emissions but is energy-intensive and costly without renewable integration [43]. For formic acid production, electrochemical reduction using Bi-based catalysts achieves >90% Faradaic efficiency but struggles with long-term stability and electrolyte management [46]. Photocatalytic systems offer modular operation but lower production rates and light-distribution limits, while thermocatalytic hydrogenation shows higher TRL and continuous operation but depends on green H_2_ and faces separation hurdles [47]. Ultimately, integrated capture–conversion systems such as calcium looping-DRM or amine–electrolysis hybrids present promising routes to bypass energy-intensive intermediate steps, yet they remain at lower TRL and require further validation under industrial conditions.

## 4. CO_2_ Conversion

The process of CO_2_ conversion involves transforming CO_2_ into useful products or fuels, aiming to reduce its environmental impact while simultaneously generating economic value. This approach is closely connected to Carbon Capture and Utilization (CCU), which focuses not only on capturing CO_2_ from industrial emissions or the atmosphere but also on converting it into marketable chemicals, fuels, or materials. Unlike simple carbon storage, CCU emphasizes the reuse of CO_2_, thus contributing to a circular carbon economy [48]. Overall, CO_2_ conversion technologies particularly into formic acid and methanol represent significant advances in CCU. They not only reduce greenhouse gas emissions but also transform CO_2_ into valuable resources, creating a bridge between environmental sustainability and economic viability. Various methods are employed for CO_2_ conversion, including chemical, biological, and electrochemical approaches [49].

### 4.1. CO_2_ Conversion into Formic Acid

Converting CO_2_ into formic acid represents an important chemical reaction, which turns CO_2_ into a valuable carboxylic acid. Formic acid is widely used in industry for different purposes, such as a preservative, antibacterial agent, coagulant, and as a starting material in chemical production [50]. This conversion typically involves metal catalysts like ruthenium, palladium, or copper to facilitate the reduction of CO_2_ to formic acid [51]. It is part of ongoing research efforts for CCU to mitigate CO_2_ emissions and offers promise for both environmental and economic reasons [52,53,54].

**Figure 4:**
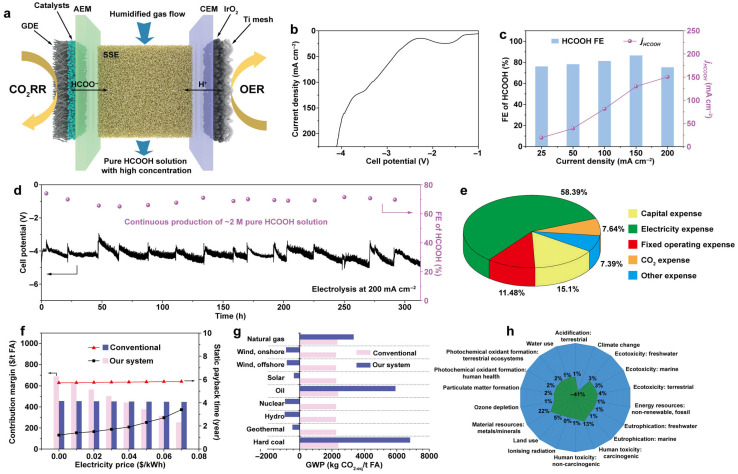
(**a**) Diagrammatic illustration of the ECO_2_RR in MEA with SSE generating a high concentration of pure HCOOH solution. (**b**,**c**) LSV curve (**b**) FE of HCOOH at different cell current densities, and associated partial current densities of HCOOH (**c**). (**d**) Long-term stability test and FEs for CO_2_ reduction to FA solution at 200 mA cm^−2^ over RD-Bi catalyst in MEA with SSE. (**e**) Used the ECO_2_RR method to divide the cost of generating 1 t FA. (**f**) ECO_2_RR offers higher contribution margins and shorter payback times than conventional methyl formate hydrolysis across varying power prices. (**g**) The potential for global warming of ECO_2_RR and conventional formate hydrolysis methods is also discussed. (**h**) ECO_2_RR outperforms conventional methyl formate hydrolysis by delivering greater profitability and faster cost recovery under different electricity prices [46].

Zhang and his colleagues [46] developed a novel approach for catalytically reducing CO_2_ to formic acid leveraging bismuth-based catalysts with lattice distortion defects, known as Bi-based catalysts with rich lattice distortion defects (RD-Bi). This was accomplished utilizing laser-irradiation in liquid-phase (LIL) to generate amorphous BiO_x_ nanoparticles, which were subsequently electrochemically reduced to RD-Bi. The method resulted in a high yield of 2 M formic acid at an industrial current density of 200 mA cm^−2^ over 300 h with Faradaic efficiency of 94.2% for formate (Figure 4b–d). Further techno-economic analysis and life cycle assessment suggest that this technique could potentially replace the traditional hydrolysis of methyl formate in commercial formic acid production. Additionally, the formic acid produced can serve as a direct fuel in air-breathing formic acid fuel cells, achieving a power density of 55 mW cm^−2^ and remarkable thermal efficiency of 20.1% (Figure 4e–h). These results underscore the promise of electrochemical CO_2_ reduction as a sustainable, cost-effective, and environmentally friendly method for producing formic acid.

The electrochemical reduction of CO_2_ to formic acid presents challenges for industrial-scale deployment, including scalability of reactor designs, uniform current distribution, mass transfer at high densities, electrolyte stability, and catalyst durability amidst flue gas impurities. Additionally, improving energy efficiency through reduced overpotentials and integration with low-carbon electricity sources is crucial for environmental and economic viability [55].

Additional practical hurdles involve the downstream separation and purification of formic acid from the electrolyte and gaseous co-products, which can significantly impact overall process energy and cost. The integration of electrochemical conversion with upstream CO_2_ capture—central to the concept of integrated carbon capture and conversion (ICCC)—also demands careful alignment of operating conditions and material compatibility to avoid efficiency losses [56]. Addressing these challenges through advances in catalyst design, reactor engineering, and process intensification will be essential for translating laboratory achievements into commercially viable, sustainable formic acid production systems [57].

Industrial methyl-formate hydrolysis typically requires 5.6–7.3 GJ per ton of formic acid and results in production costs of 450–650 USD per ton. In contrast, the RD-Bi electrochemical CO_2_-to-formic acid system operates at 3.2–3.8 V, equivalent to an energy demand of 8–11 MWh per ton of formic acid, and shows competitive economic performance under renewable-electricity scenarios. These quantitative comparisons highlight the potential economic advantages of RD-Bi technology relative to conventional processes.

**Figure 5:**
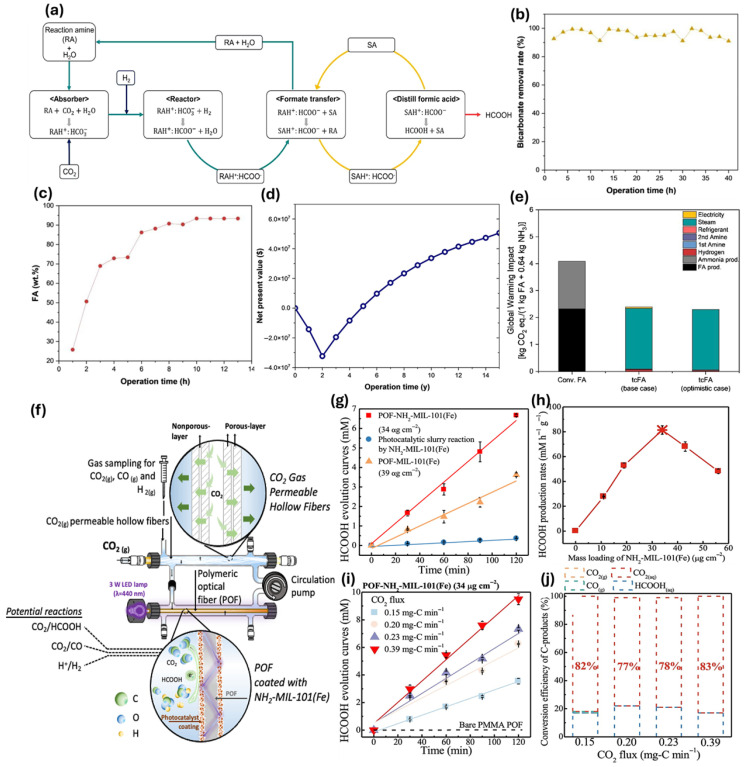
(**a**) Illustration of the formic acid (FA) production process through CO_2_ hydrogenation, with colored streamlines indicating reaction and separation amines (cyan), input materials (yellow), and FA output (red), and bicarbonate flow (navy). key performance indicators (KPIs) (**b**) for bicarbonate removal and (**c**) the amount of FA recovered through distillation. (**d**) Net present value analysis indicating the process’s economic viability. (**e**) Global warming impact indices [47]. (**f**) Dual-fiber reactor with NH_2_-MIL-101(Fe)-coated optical fibers and hollow fibers enables photocatalytic CO_2_ reduction to formic acid. (**g**) Comparison of HCOOH yields under 440 nm LED using NH_2_-MIL-101(Fe)-coated POFs, MIL-101(Fe), and NH_2_-MIL-101(Fe) slurry. (**h**) Optimal catalyst surface loading determined at 34 μg cm^−2^ NH_2_-MIL-101(Fe). (**i**) CO_2_ flux results and (**j**) carbon mass balance after 2 h of photocatalytic CO_2_ reduction shows efficiency trends with optimized catalyst loading under different CO_2_ pressures [58].

A new cost-effective and efficient method for producing formic acid from CO_2_ has been developed by Kim et al. [47], which has potential environmental benefits, and could help achieve a net-zero economy in the face of the climate crisis. This study presents an innovative CO_2_ hydrogenation process for producing formic acid (Figure 5a) at a pilot scale of 10 kg/day, achieving 92 wt% purity. The process demonstrates a 37% cost reduction and a 42% decrease in global warming impact compared to conventional methods, validated by continuous operation for over 100 h with an 82% CO_2_ conversion rate (Figure 5a,b). Key strategies include optimal amine selection and corrosion mitigation, enhancing operational efficiency. Techno-economic analysis indicates a net present value of $5.1 million over 15 years (Figure 5d), while life cycle assessment reveals a 41.5% reduction in global warming potential (Figure 5e), showcasing significant environmental benefits.

As shown in Figure 5f, Wang et al. [58] presented a novel dual-fiber reactor system for efficient CO_2_ conversion to formic acid (HCOOH) using polymeric optical fibers (POFs) coated with NH_2_-MIL-101(Fe) and hollow-fiber membranes (HFMs) for bubble-free CO_2_ delivery. This system achieved a remarkable production rate of 116 ± 1.2 mM h^−1^ g^−1^ (Figure 5h), with a quantum efficiency of 12% based on the HCOOH generation (Figure 5g), significantly outperforming traditional slurry-based methods. The dual-fiber design enhanced light utilization and CO_2_ adsorption, achieving 99% selectivity for HCOOH. Figure 5i shows that the peak production rate of formic acid (HCOOH) was 116 mM h^−1^ g^−1^ (k = 3.5 × 10^−4^ mM s^−1^), occurring at a CO_2_ pressure of 10 psig. This rate is over 2.7 times higher than the production rate observed at 2 psig (k = 1.9 × 10^−4^ mM s^−1^). Furthermore, the greatest carbon-conversion efficiency of CO_2_ to HCOOH was attained at 3 psig of CO_2_, recorded at 0.2 mg-C·min^−1^ (Figure 5j).

### 4.2. CO_2_ Conversion into Methanol

Converting CO_2_ to methanol is an attractive process to reduce emissions and produce valuable chemical feedstock [59]. The process involves capturing CO_2_, producing hydrogen, combining CO_2_ and H_2_ to make methanol, purifying the methanol, and using it as fuel, solvent, or feedstock [60]. Challenges include the energy-intensive nature of capturing and purifying CO_2_, and the need for efficient catalysts [61,62,63]. Continuing research and development efforts are focused on optimizing the efficiency and economic viability of converting CO_2_ into methanol as a strategy to mitigate climate change [64].

**Figure 6:**
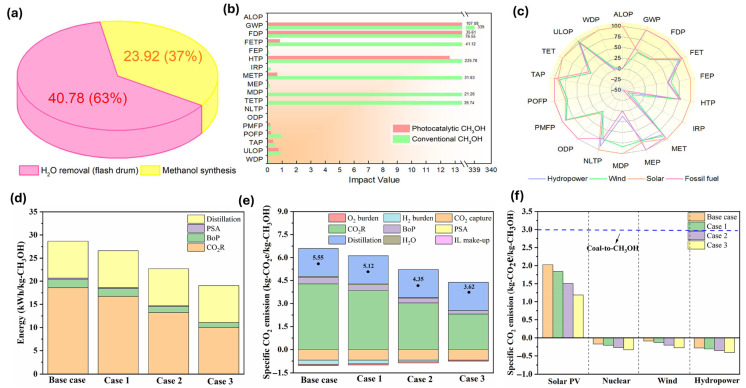
(**a**) Detailed energy usage in kWh for the CH_3_OH synthesis step using the traditional equipment. (**b**) Overview of the 18 midpoint indicator analysis. (**c**) Sensitivity analysis of replacing fossil fuels with renewables. The impact ratings are adjusted to the greatest value of each indicator as a percentage (%) [65]. (**d**) Energy and (**e**) specific CO_2_ emissions for CO_2_R under different scenarios. (**f**) Specific CO_2_ emissions for CO_2_R under different scenarios with 100% utilization of solar photovoltaic (PV) [66].

Sheng Ling et al. [65] assessed a photocatalytic CO_2_ reduction system utilizing graphitic carbon nitride (g-C_3_N_4_) for methanol production. They demonstrated a 68% decrease in carbon footprint and a 53% reduction in fossil fuel consumption compared to conventional steam methane reforming. Key results highlight lower energy requirements (Figure 6a) and notable environmental advantages (Figure 6b). Sensitivity analysis suggests that incorporating renewable energy, particularly hydropower, can further lessen environmental impacts (Figure 6c). This study underscores the promising role of photocatalytic technology in enabling sustainable, industrial-scale methanol production.

Li and colleagues [66] evaluated the economic and environmental feasibility of methanol production via electrochemical reduction of CO_2_ from bio syngas. They highlight that investment and electricity costs are significant contributors to total production costs (TPC), with integrated systems reducing TPC by 28–66% (Figure 6d,e). Current stand-alone systems are economically unviable, but future advancements could lower costs to 0.50 €/kg-CH_3_OH. Renewable energy sources can enhance sustainability, achieving negative specific CO_2_ emissions (Figure 6f). The study emphasizes the importance of integrating CO_2_R with biomass gasification to improve economic competitiveness and environmental benefits.

**Figure 7:**
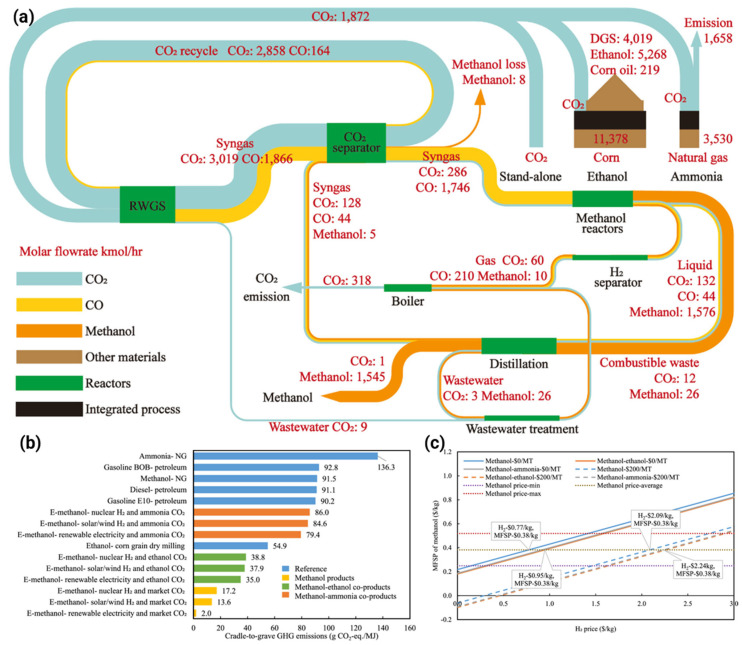
The study examines the CO_2_ conversion and specific electricity consumption of: (**a**) Sankey diagram illustrates carbon flow balances (kmol/h) across components, processes, and material streams in three systems. (**b**) CTGR analysis compares synthetic methanol’s GHG emissions with other fuels using renewable H_2_ sources and U.S. grid-based methanol synthesis. (**c**) Breakeven H_2_ costs for various CO_2_ credit values and methanol production systems are also presented [67].

Zang et al. [67] analyzed the technoeconomic and life cycle aspects of synthetic methanol production using renewable H_2_ and high-purity CO_2_ from ethanol and ammonia plants. It identifies three production systems: integrated methanol–ethanol, integrated methanol-ammonia, and standalone methanol coproduction. The stand-alone system achieves cradle-to-grave emissions of 13.6 g CO_2_-equiv/MJ, significantly lower than conventional methanol at 91.5 g CO_2_-equiv/MJ (Figure 7b). Carbon credit can further reduce costs (Figure 7c). The study highlights that integrating methanol synthesis with other processes improves efficiency, with a carbon conversion efficiency of 82.5% and energy efficiency of 75.6% (Figure 7a). The findings suggest broader applications across various industries to enhance environmental benefits.

The electrochemical route for CO_2_ conversion to formic acid is inherently more suitable for decentralized and modular applications, whereas the hydrogenation pathway is better aligned with centralized, large-scale production. This distinction arises from fundamental differences in energy input, system complexity, and infrastructure requirements. Electrochemical CO_2_ reduction operates under relatively mild temperatures and pressures and can be directly powered by renewable electricity. In contrast, hydrogenation requires a continuous hydrogen supply and large-scale setups, favoring centralized operations with economies of scale but limiting decentralized use. The trade-off is between scalability and flexibility, with electrochemical systems offering operational advantages but facing challenges like electricity cost and catalyst durability, while hydrogenation is more mature but dependent on centralized infrastructure. Both pathways should be seen as complementary, serving different segments of CO_2_ utilization.

In Table 2, the summary presents CO_2_ conversion routes, focusing on their scalability and readiness for producing bulk fuels and intermediates. Routes that generate urea, syngas, and methanol show higher scalability and TRLs, making them suitable for centralized industrial use, with efficiencies of 50–70%. However, they require significant renewable hydrogen and effective CO_2_ integration. In contrast, electrochemical processes that produce carbon monoxide and formic acid are less mature, indicated by lower TRLs, but allow for decentralized operations and better alignment with renewable energy. The choice of product impacts conversion strategy and reactor design, with various CO_2_ conversion technologies having different maturities and efficiencies for fuels, chemicals, and building materials.

Formic acid, produced via electrochemical reduction, represents a promising liquid energy carrier and chemical intermediate. While its Faradaic efficiencies are high at lab scale (40–60%), broader deployment is currently restricted by catalyst stability and high energy costs. Carbon monoxide serves as a key feedstock for downstream chemical synthesis, yet challenges in selectivity and catalyst durability limit its widespread use. Industrial-scale urea production is already commercialized (TRL 8–9) and achieves high efficiency (60–70%), but its environmental benefits depend on replacing conventional ammonia with green hydrogen. Polymers and carbonates, synthesized via chemical fixation, offer high conversion efficiency (60–90%), though their applicability is constrained by smaller markets and catalyst costs. Mineralization, which converts CO_2_ into stable Ca/Mg carbonates for construction, combines high efficiency (70–90%) with very high scalability (TRL 7–9), providing a permanent and environmentally beneficial route, despite slow reaction kinetics and the energy required for mineral processing.

Overall, methanol and mineralization pathways appear most promising for large-scale deployment, whereas formic acid, syngas, and polymers serve as niche or transitional solutions. The success of all CO_2_ conversion routes depends critically on integration with renewable energy sources and efficient coupling with capture technologies, emphasizing the need for system-level optimization in sustainable carbon management strategies.

### 4.3. Molecular Mechanisms and Active Sites in Catalytic CO_2_ Conversion

Advancing CO_2_ conversion technologies requires a molecular-level understanding of the catalytic cycles involved. In thermocatalytic methanol synthesis, Cu/ZnO/Al_2_O_3_ catalysts typically follow a formate pathway, where CO_2_ is sequentially hydrogenated via adsorbed formate and methoxy intermediates. Catalytic activity is closely tied to the Cu–ZnO interface and the oxidation state of Zn, with the hydrogenation of formate often being rate-limiting [68]. In electrocatalytic CO_2_ reduction (CO_2_RR), product selectivity hinges on the catalyst’s electronic structure and interactions with intermediates. On Cu surfaces, *CO dimerization dictates multi-carbon product formation, influenced by local pH, electric field, and crystal facet. For formate-selective metals like Sn or Bi, stabilization of the *OCHO intermediate is critical, though catalyst degradation under operating conditions remains a challenge [69].

In photocatalytic systems, efficiency depends on the cascade from photon absorption to surface redox reactions, with charge recombination being a major bottleneck. Modifications such as nitrogen vacancies in g-C_3_N_4_ or heterojunction formation can enhance charge separation, while coordinatively unsaturated metal sites in MOF-based photocatalysts activate CO_2_ by stabilizing key reduction intermediates like *COOH.

## 5. Integrated Carbon Capture and Conversion/Utilization (ICCC/ICCU)

Integrated Carbon Capture and Conversion/Utilization (ICCC/ICCU) refers to a class of technologies that combine the capture of CO_2_ emissions with their immediate conversion or utilization into valuable products within a single, often synergistic system. ICCC/ICCU technologies aim to enhance the economic efficiency of CO_2_ conversion by addressing energy-intensive processes, such as purification and compression [70].

### Molecular Synergies in Dual-Function Materials (DFMs) for ICCU

Integrated carbon capture and conversion utilizes Dual-Function Materials (DFMs), which simultaneously capture CO_2_ and catalyze its conversion. For instance, in CaO–Ni DFMs, CaO captures CO_2_ as CaCO_3_, which then decomposes in a CH_4_ atmosphere to release CO_2_ at Ni nanoparticle sites, enhancing syngas yield and reducing equilibrium limitations. Strong metal–support interactions at the Ni–CaO interface increase catalyst activity and coking resistance, while CeO_2_ aids in stabilizing Ni and promoting carbon removal. Similarly, in amine-supported systems with metals like Pd and Ru, CO_2_ converts to carbamate/bicarbonate and spills over to catalytic sites for reduction. Challenges include the kinetics of spillover and amine stability under reducing conditions, with DFT and in situ spectroscopy assisting in understanding interfacial processes and designing efficient DFMs [32,71,72].

**Figure 8:**
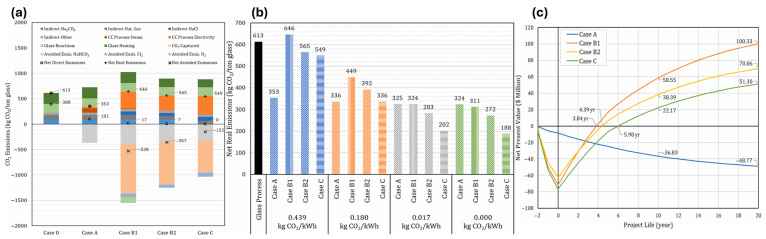
(**a**) A summary of positive and negative emissions from the glass process and four potential carbon capture technologies. The paper highlights that instances 0 and A have no averted emissions. (**b**) Shows how four carbon capture methods are related by analyzing their net CO_2_ emissions and how sensitive they are to electrical CO_2_ emission parameters. (**c**) Net present values for the four carbon capture technologies during a 20-year project life cycle [73].

Caudle et al. [73] investigated the ICCU in the glass industry to reduce CO_2_ emissions, emphasizing the economic and environmental benefits of CO_2_ mineralization. It models three cases (B1, B2, C) against a conventional CCS case (A). Case A, utilizing amine absorption, captures 380 kg CO_2_/ton of glass but lacks profitability without carbon credits. In contrast, Case B1 captures the same amount while producing 127 kton/year of NaHCO_3_ and additional byproducts, achieving net negative emissions of −528 kg CO_2_/ton glass. Case B2 partially replaces natural gas with H_2_, reducing emissions to 338 kg CO_2_/ton, while Case C incorporates Na_2_CO_3_ recycling, capturing 100% of CO_2_ but requiring higher capital investment. The analysis highlights that all CCU cases demonstrate significant emissions reduction and profitability, illustrating emissions comparisons (Figure 8a), net real (Figure 8b), and net present values (Figure 8c), respectively, underscoring the potential for strategic application in hard-to-abate industries.

**Figure 9:**
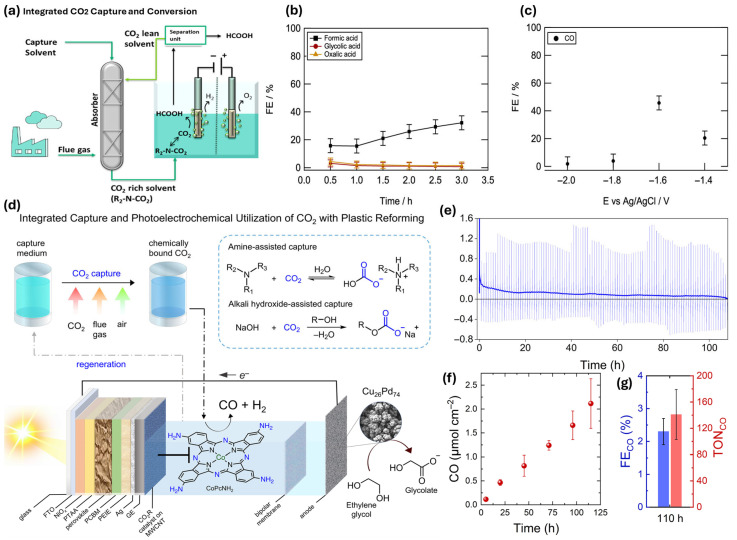
Illustration of integrated CO_2_ capture and conversion strategies, including: (**a**) CO_2_ absorption in amine solution (carbamate/bicarbonate forms). (**b**,**c**) Faradaic efficiencies for formate, glycolic acid, oxalate (Pb electrode) and CO (Au electrode) under varying electrolysis conditions [74]. (**d**) Using solar energy, the one-step PEC process upcycles ethylene glycol from plastic to glycolic acid and turns post-capture solution into syngas. (**e**–**g**) PEC performance over 110 h: (**e**) photocurrent stability, (**f**) linear CO production, and (**g**) turnover number (TON) with Faradaic efficiency, demonstrating long-term system stability under simulated solar irradiation [75].

Pérez-Gallent et al. [74] present an integrated approach for CO_2_ capture and electrochemical conversion using amine-based solvents as electrolytes (Figure 9a). This method eliminates the energy-intensive CO_2_ desorption step, enhancing economic feasibility and efficiency. At elevated temperatures (75 °C), formate production rates increase significantly, achieving faradaic efficiencies of about 40% (Figure 9b). The use of a mixture of 2-amino-2-methyl-1-propanol (AMP) and propylene carbonate (PC) improves efficiency, with 1 M AMP concentration optimizing faradaic efficiency despite potential mass transfer limitations. Validated in a semi-continuous flow reactor, the system shows promise for large-scale applications, with gold electrodes yielding up to 45% faradaic efficiency for carbon monoxide production (Figure 9c). This integrated methodology supports the advancement of a circular carbon economy.

Kar et al. [75] discuss an innovative system for capturing and converting CO_2_ from various sources in their article, including concentrated streams, flue gas, and ambient air, into syngas using solar energy (Figure 9d). The process employs amine or hydroxide solutions for efficient CO_2_ capture, facilitated by a cobalt-phthalocyanine catalyst. This integrated approach allows for direct conversion of captured CO_2_ into syngas, thus addressing both carbon utilization and waste valorization. Figure 9e shows the photocurrent transients over a 110 h experiment, which exhibit fluctuations due to charge recombination. In contrast, Figure 9f illustrates a linear increase in CO production, demonstrating the system’s consistent performance. The experiment achieved a faradaic efficiency of 2.3% and a turnover number (TON) of 141 for CO production, highlighting the effectiveness of converting atmospheric CO_2_ into usable products, as shown in Figure 9g.

Table 3 provides an overview of current integrated carbon capture and conversion/utilization (ICCC/ICCU) strategies, comparing different approaches in terms of capture–conversion integration, energy requirements, efficiency, technology readiness, and practical advantages. Among the methods, chemical looping CO_2_ capture and in situ conversion and mineralization via integrated absorption stand out due to their high overall efficiency (60–90%), energy efficiency, and relatively advanced maturity (TRL 6–7 for chemical looping, TRL 7–9 for mineralization). These systems offer compact designs, reduce intermediate energy costs, and enable either permanent or value-added CO_2_ utilization, making them highly promising for industrial-scale deployment. Other approaches, such as absorption-methanolization and membrane-coupled electrocatalysis, provide the advantage of directly producing fuels or chemicals, but they face challenges including high renewable hydrogen demand, catalyst stability, and scale-up limitations [71]. Photocatalysis- and ionic liquid-based adsorption systems offer modular and low-energy options suitable for solar-driven processes, yet they are still at early research stages (TRL 4–5) and require further development for commercial application. Overall, the choice of ICCC/ICCU method depends on the intended application: mineralization and chemical looping are preferable for permanent CO_2_ storage and large-scale industrial integration, while absorption-methanolization and membrane-electrocatalysis are more suitable for producing fuels or high-value chemicals when renewable energy and hydrogen are available. Emerging photocatalytic and high-value adsorption systems show potential for decentralized or off-grid applications. In summary, high TRL systems with integrated capture and conversion, such as chemical looping and mineralization, are currently the most feasible for near-term deployment, whereas other technologies are expected to become competitive as renewable energy integration and catalyst performance advance. Thus, chemical looping and mineralization represent the most practical and scalable ICCC/ICCU strategies for immediate industrial implementation [76].

## 6. Conclusions

ICCU technologies represent a shift towards more efficient carbon management by turning captured CO_2_ into useful products as compared with traditional CCU technologies. This approach aims to combat climate change by creating closed-loop systems. However, success relies on overcoming various challenges, including technical, regulatory, economic, and societal issues, which require collaboration among researchers, industries, and policymakers. Recent improvements in photocatalytic and photoelectrochemical technologies have focused on increasing efficiency and stability under real sunlight. It is important to design effective materials for these systems, particularly for applications like treating flue gas. This involves choosing engineering photocatalysts to improve their performance and durability against harsh conditions. Dual-function materials are noteworthy because they can capture and convert pollutants at the same time, enhancing performance and sustainability. Their design aims to resist damage from heat and chemicals. According to this review, integrating ICCU technologies with cutting-edge material design is essential for creating a sustainable, low-carbon economy to lower the cost of ICCU technologies. Additionally, achieving this economic model necessitates a combination of financial incentives, supportive regulations, and international collaboration.

## Figures and Tables

**Table 1:** Comparative Insights into CO_2_ Capture Technologies.

Capture Method	Typical Process/Sorbent	Capture Efficiency	Energy Demand	Scalability Potential	TRL *	Key Challenges
**Amine-based Absorption**	Aqueous amines (e.g., MEA, DEA)	85–95%	High (2–4 GJ/ton CO_2_)	Very High (commercial scale in power plants)	8–9	Solvent degradation, corrosion, high regeneration cost
**Solid Sorbents**	Zeolites, MOFs, activated carbons	70–90%	Moderate (1–2.5 GJ/ton)	High (modular design possible)	5–7	Moisture sensitivity, limited long-term stability
**Membrane Separation**	Polymeric, ceramic, hybrid membranes	60–85%	Moderate (depends on pressure)	Moderate to High	5–7	Selectivity vs. permeability trade-off, scaling cost
**Cryogenic Separation**	Cooling & liquefaction of CO_2_	>95%	Very High (>5 GJ/ton)	Low–Moderate	4–6	Energy-intensive, suitable mainly for high CO_2_ streams
**Calcium Looping**	CaO/CaCO_3_ cycle (high-temp sorbent)	85–95%	High (3–4 GJ/ton)	High (integrates with power plants)	6–7	Sorbent sintering, large reactor footprint
**Direct Air Capture (DAC)**	Solid sorbents or alkaline solutions	50–70%	Very High (5–8 GJ/ton)	Moderate (scaling under development)	4–6	High cost ($200–600/ton), infrastructure needs
**Biological Capture**	Algae cultivation, biochar	Variable (30–70%)	Low–Moderate	Moderate (depends on land/water use)	3–5	Land footprint, low CO_2_ uptake rate

* TRL: Technology Readiness Level (1 = basic research, 9 = fully commercial).

**Table 2:** Comparative Summary of Major CO_2_ Conversion Pathways.

Product	Main Conversion Method(s)	Energy Source/Input	Typical Efficiency	Scalability Potential	TRL *	Key Challenges
**Formic Acid (HCOOH)**	Electrochemical reduction, homogeneous catalysis	Renewable electricity, H_2_	40–60% (lab scale)	Moderate (chemical feedstock, fuel cell use)	4–5	Catalyst stability, high energy cost
**Methanol (CH_3_OH)**	Catalytic hydrogenation, electrochemical	H_2_ from renewable electrolysis	50–70%	High (fuels, plastics, chemicals)	6–7	High green H_2_ demand, need CO_2_ capture
**Syngas (CO + H_2_)**	Reverse water–gas shift (RWGS), plasma catalysis	High-temp heat + H_2_	~50%	Very High (precursor for fuels/chemicals)	6–7	Energy-intensive, requires efficient H_2_
**Carbon Monoxide (CO)**	Electrochemical CO_2_ reduction, RWGS	Renewable electricity, H_2_	40–60%	High (intermediate for Fischer–Tropsch, methanol)	5–6	Selectivity, catalyst degradation
**Urea**	Reaction with NH_3_ (from Haber–Bosch)	CO_2_ + NH_3_ (energy-intensive)	60–70%	High (fertilizer industry)	8–9	NH_3_ needs green H_2_
**Polymers/Carbonates**	Chemical fixation (epoxides, alcohols)	Mild heat, catalysts	High (60–90%)	Moderate (niche polymers)	5–6	Market size limited, catalyst cost
**Mineralization (Ca/Mg Carbonates)**	Direct reaction with minerals/industrial wastes	Heat, pressure (sometimes ambient)	70–90%	Very High (cement, construction)	7–9	Slow kinetics, energy for mineral processing

* TRL: Technology Readiness Level (1 = basic research, 9 = commercial deployment).

**Table 3:** Overview of Integrated Carbon Capture and Conversion/Utilization (ICCC/ICCU) Technologies.

System Type	Main Approach	Energy/Input	Typical Efficiency	TRL	Advantages	Key Challenges
**Integrated Absorption & Mineralization**	CO_2_ absorbed by amines + mineral reaction	Heat, solvent, CaO	70–90%	7	Permanent CO_2_ storage, scalable, low cost	Slow kinetics, solvent degradation, mineral processing energy
**Integrated Absorption & Methanolization**	CO_2_ captured in amines → methanol	Renewable H_2_, heat, catalyst	50–70%	6	Produces fuel/chemical directly, integrated process	High H_2_ demand, catalyst/separation issues
**Integrated Membrane & Thermocatalysis**	CO_2_-permeable membrane + catalytic conversion	Heat, membrane, catalyst	40–60%	5–6	Compact, continuous operation	Membrane stability, CO_2_ flux, scale-up
**Integrated Membrane & Electrocatalysis**	Membrane separation + electrochemical CO_2_ reduction	Renewable electricity, H_2_	40–60%	5	Modular, renewable-powered	Low CO_2_ concentration, Faradaic efficiency, catalyst durability
**Integrated Adsorption & Photocatalysis**	Adsorbents + light-driven conversion	Solar light, adsorbents	30–50%	4–5	Solar-driven, modular	Limited light absorption, low scale, catalyst degradation
**Integrated Adsorption & High-Value Conversion**	Ionic liquids capture + selective conversion	Heat, catalysts, ILs	40–60%	4–5	High selectivity, dual-use of CO_2_	High IL cost, selectivity, scale-up
**Chemical Looping CO_2_ Capture & In Situ Conversion**	Redox solids capture + catalytic conversion	Heat, catalyst, reactive solids	60–80%	6–7	Energy-efficient, compact, integrated	Material degradation, reactor integration, scaling

TRL: Technology Readiness Level (1 = basic research, 9 = commercial deployment).

## Data Availability

No new data were created or analyzed in this study. Data sharing is not applicable to this article.
